# Megavoltage Radiotherapy for the Treatment of Degenerative Joint Disease in Dogs: Results of a Preliminary Experience in an Italian Radiotherapy Centre

**DOI:** 10.3389/fvets.2018.00074

**Published:** 2018-04-10

**Authors:** Federica Rossi, Simona Cancedda, Vito Ferdinando Leone, Carla Rohrer Bley, Paola Laganga

**Affiliations:** ^1^Centro Oncologico Veterinario, Sasso Marconi, Italy; ^2^Clinica Veterinaria dell’Orologio, Sasso Marconi, Italy; ^3^Division of Radiation Oncology, Vetsuisse Faculty, University of Zurich, Zurich, Switzerland

**Keywords:** low-dose radiotherapy, osteoarthritis, degenerative joint disease, dog, canine

## Abstract

The aim of the study was to evaluate the efficacy and toxicity of a low-dose radiotherapy treatment in dogs with osteoarthritis (OA). Inclusion criteria were dogs affected by OA of one or multiple joints, with lameness, previously treated with medical therapy and referred for radiotherapy because of chronic unresponsive pain. After suspension of medical therapy, dogs underwent external beam radiotherapy treatments delivered in three fractions of 2 Gy each. Four of these dogs had one (three dogs) to four (one dog) additional courses of radiation. Medical records were reviewed and follow-up information was collected by clinical recheck and owners interview. Twenty-five dogs matched the inclusion criteria; among them, 21 had one course of RT and 4 underwent multiple treatments, respectively 218, 266, 39, and 1,384 days after the first treatment. Clinical improvement was observed in 92% of patients with median benefit duration of 356 days after the first treatment, and 418 days after the second treatment. No side effects were recorded. In this group of patients, radiotherapy was effective, well tolerated, and repeatable, leading to an improvement of quality of life in dogs with degenerative joint disease unresponsive to medical treatments.

## Introduction

In dogs, osteoarthritis (OA) is a frequent, often under-diagnosed and non-adequately treated chronic disease (1). OA is a progressive joint disease characterized by pain, reduced joint range of motion (ROM), cartilage destruction, and bone remodeling (2). Inflammation of the synovial cells and chondrocytes dysfunction are responsible for the biochemical changes behind this condition, with subsequent release of cytokines, free radicals and prostaglandins, reduced proteoglycans production, and increased lysosomal enzyme production (2, 3). Vasodilation, increased vascular permeability, and pain receptor stimulation result in joint swelling, increased local temperature, crepitus, and pain (2). Disease chronicity is characterized by muscular atrophy and decreased ROM (2). Radiographic changes include soft tissue thickening due to joint effusion and capsular fibrosis, subcondral bone sclerosis, and periarticular osteophytes (2).

The goal of OA treatment is to decrease joint effusion and cartilage damage, slower the fibrotic process and finally control pain, and is based on the combination of medical therapy, weight control, nutritional supports, and physical rehabilitation (1, 2, 4, 5). Non-steroid and steroid anti-inflammatory drugs and opiates are often used in dogs affected by OA, alone or in combination, to control clinical symptoms; however, they are often insufficient to provide adequate mobility and good quality of life (QoL) and are frequently associated with gastrointestinal and renal side effects if used in the long period (6, 7). Other innovative treatments, including autologous platelet therapy and mesenchymal cells administration, have been proposed in veterinary medicine with encouraging preliminary results (8–11).

Low-dose radiation therapy (LDRT) is a well-known treatment in human medicine to control chronic unresponsive pain in the long term; partial or complete responses in patients with painful arthrosis have been reported in 24–100% of treated cases, depending on the study (12–14). The radiobiological mechanism in inflammatory diseases of LDRT has been investigated and confirmed, however, not completely understood (15, 16). The anti-inflammatory and analgesic effect is the result of a combination of cell activity modulation (reduced adhesion of blood mononuclear cells to endothelial cell, macrophages, and granulocytes activity regulation) and anti-inflammatory factors production (lowered production of cytokine, nitric oxide, and reactive oxygen species) (16). Similar clinical results are reported in horses, where a dose raging between 5 and 15 Gy has been used to treat degenerative joint diseases (DJD) (17, 18). First experiences of local radiation therapy by intra-articular administration of β-emitting radionuclides are reported in dogs, showing improvement in clinical signs and no significant side effects (19, 20). Recently, a single-fraction 10 Gy RT protocol has been investigated in five Labrador Retrievers affected by elbow OA, with short-term clinical benefit and improved weight bearing in the affected limb (21). However, there is no similar information on long-term external-beam RT effect in different diseased joints in dogs.

The aim of this retrospective study was to evaluate the response to LDRT in a cohort of dogs affected by DJD of single or multiple joints after interruption of medical therapy. It was hypothesized that RT as single treatment modality would provide an improvement in lameness severity and QoL.

## Materials and Methods

### Inclusion Criteria

Dogs affected by OA of one or multiple joints presented to the Centro Oncologico Veterinario between May 2009 and December 2016 and treated with LDRT were retrospectively included in the study. Dogs had to be affected by various grades of lameness, visited by a veterinarian orthopedic, having radiographic evidence of OA, and being unresponsive to drugs or other types of treatments. Medical records were reviewed to retrieve demographic information [breed sex, age, weight, and body condition score (BCS)], results of orthopedic evaluation, and clinical examination at the RT center, previous medical and non-medical treatments, results of blood tests, and radiographic examination. Lameness location during walking and trotting, lameness (grades 1–5) and evaluation of pain during palpation (grades 1–5) were recorded (Table 1) using previously validated grading systems (22, 23).

**Table 1:** Lameness and pain score system used to assess response to low-dose radiation therapy (22, 23).

Category and score	Clinical signs
**Lameness**	
1	Stands, walks, and trots normally
2	Stands normally, slight lameness under special circumstances or inconsistent lameness when trotting
3	Stands normally, obvious lameness when trotting, consistent lameness when walking
4	Abnormal position while standing, obvious lameness under any circumstances
5	Reluctant to rise and walk, intermittent or continuous non-weight-bearing lameness

**Pain on palpation**	
1	No signs of pain during palpation of affected joint
2	Mild pain during palpation; dog turn heads in recognition
3	Moderate pain during palpation; dog pulls limb away
4	Severe pain during palpation; dog vocalizes or becomes aggressive
5	Dog will not allow examiner to evaluate the joint

### RT Treatment

Dogs were treated under general anesthesia in dorsal, sternal, or lateral recumbency, depending on the anatomic location of the affected joint(s), with electron beam radiation delivered using a linear accelerator (Clinac DMX; Varian Medical Systems, Palo Alto, CA, USA). Treatment planning was performed by hand calculation. Radiographs of the joint taken in two orthogonal projections were used to calculate the thickness of the joint and the field size, in order to include the entire joint in the treatment field. The prescribed dose was 6 Gy divided in three fractions of 2 Gy each over three consecutive days or on an alternate scheduling (Monday, Thursday, Friday), delivered with a single 9–15 MeV electron field. The 90% isodose line was chosen to encompass this target volume and for dose normalization (24). Source–surface distance was set at 100 cm.

Treatment response was evaluated by a clinical recheck 3 weeks after the end of RT, and repeated every 3 months thereafter. Partial response was defined as lameness improvement of at least one grade, complete response was considered if the dog was free of lameness at the 3 weeks rechecks. Before the treatment and 3 weeks after the end of RT, owners were asked to answer a chronic pain index questionnaire. The questionnaire was in Italian and included 11 questions with 5 answers each based on a 5 points descriptive scale, testing animal’s behavior and locomotion, as previously described (25) (Table 2) The answer to those questions were assigned index scores from 0 to 4. The total chronic pain index score ranged from 0 to 44, where 0 represented lack of pain (normal) and 44 the highest pain level. Median score for dogs before and after treatment was calculated and compared through the Wilcoxon matched-pairs signed rank test. Treatment toxicity was evaluated and graded based on the Veterinary Radiation Therapy Oncology Group Criteria (26).

**Table 2:** Pain assessment questionnaire including 11 questions with 5 answers (19).

Assessed item	0	1	2	3	4
Mood	Very alert	Alert	Neither alert nor indifferent	Indifferent	Very indifferent
Dog’s willingness to play and games	Very willingly	Willingly	Reluctantly	Very reluctantly	Does not at all
Vocalization (audible complaining)	Never	Hardly ever	Sometimes	Often	Very often
Dog’s willingness to walk	Very willingly	Willingly	Reluctantly	Very reluctantly	Does not at all
Dog’s willingness to trott	Very willingly	Willingly	Reluctantly	Very reluctantly	Does not at all
Dog’s willingness to gallopp	Very willingly	Willingly	Reluctantly	Very reluctantly	Does not at all
Dog’s willingness to jump	Very willingly	Willingly	Reluctantly	Very reluctantly	Does not at all
Dog’s ease in lying down	With great ease	Easily	Neither easily nor with difficulty	With difficulty	With great difficulty
Dog’s ease in getting up	With great ease	Easily	Neither easily nor with difficulty	With difficulty	With great difficulty
Dog’s difficulty in movement after rest	Never	Hardly ever	Sometimes	Often	Very often or always
Movement after major exercise	Never	Hardly ever	Sometimes	Often	Very often or always

*Owners were asked to score dog’s pain from 0 to 4 before and 3 weeks after RT treatment. Differences in the lameness and pain scores at baseline and after RT were assessed with Wilcoxon matched-pairs signed rank test*.

## Results

### Demographic and Clinical Characteristics

Twenty-five dogs met the inclusion criteria. Represented breeds were Labrador Retriever (*n* = 10, 40%), German Shepherd (*n* = 5, 20%), mixed breed (*n* = 4, 16%), Rottweiler and Golden Retriever (*n* = 2 each, 8%), Great Dane and Bernese Mountain Dog (*n* = 1 each, 4%). Males were over-represented (*n* = 18, 72%, 12 intact and 6 castrated males) compared to females (*n* = 7, 48% 2 intact and 5 spayed females). Mean age was 8.9 years (median 9 years; range 2–15 years), mean weight 33.4 kg (median 32 kg; range 20–74 kg). The BCS was normal in 15 dogs (60%; BCS of 4–5/9), whereas it was increased in the remaining 10 (40%; BCS 7–9/9).

Clinically, 6 dogs had a single joint affected by OA (4 elbows and 2 stifles). The other 19 dogs had multiple (2–5) joints OA, but there was one primary affected joint, with marked soft tissue thickening, decreased ROM and pain score graded from 3 to 4. In these dogs, lameness grade and pain score were described for the most severely affected joint only. Lameness score was 4 in the majority of cases (*n* = 13, 52%), followed by 3 (*n* = 7, 28%), 5 (*n* = 3, 12%), and 2 (*n* = 2, 8%). Pain score was graded as follows: 4 in 11 dogs (44%), followed by 3 in 9 dogs (36%), 2 in 3 dogs (12%), and 1 in 2 dogs (8%).

Before RT, all dogs were treated as follows: 22 dogs (88%) received medical therapy [*n* = 17 non-steroid anti-inflammatory drugs (NSAID), *n* = 5 steroids, *n* = 8 opiates], 20 (80%) nutraceutical, and 3 (12%) dogs underwent physiotherapy. Twenty-three dogs (92%) had combined treatments. At presentation, 13 dogs (52%) were treated with nutraceutical, 5 (20%) with NSAID, and 3 (12%) with physiotherapy. In 10 dogs (40%), NSAID therapy was interrupted because of gastrointestinal side effects. All treatments were discontinued at the beginning of RT.

Radiographs of the affected joints and thorax, complete blood cell count (CBC), and serum biochemistry profile were available for all dogs. Radiographs were evaluated by single radiologist (Federica Rossi). Thoracic radiographs were within normal limits in 9 dogs (36%), whereas in 16 dogs (64%), a diffuse, mild interstitial lung pattern was observed, compatible with dog’s age and BCS. Joint radiographs showed peri-articular osteophytosis (*n* = 60 joints, 94%), subcondral bone sclerosis (*n* = 49 joints, 77%), peri-articular soft tissue thickening (*n* = 46 joints, 72%), and thinning of the joint space (*n* = 54 joints, 84%), CBC was normal in 23 dogs (92%), whereas in two cases, there was a mild normocytic anemia, and in one dog, mild neutrophilic leukopenia. Biochemistry profile showed abnormalities in 12 dogs (48%), including elevated renal parameters (urea, creatinine, and phosphor) in 4 dogs, and increased liver enzymes (ALT, AST, SAP, and γGT) in 9 dogs.

### Treatment

General anesthesia was inducted with intravenous injection of propofol at the dosage of 2–4 mg/kg (Proposure^®^ 10 mg/ml, Merial Italia spa, Milano) and maintained after intubation with a oxygen-isofluorane mixture with 2–3% isofluorane concentration.

**Figure 1:**
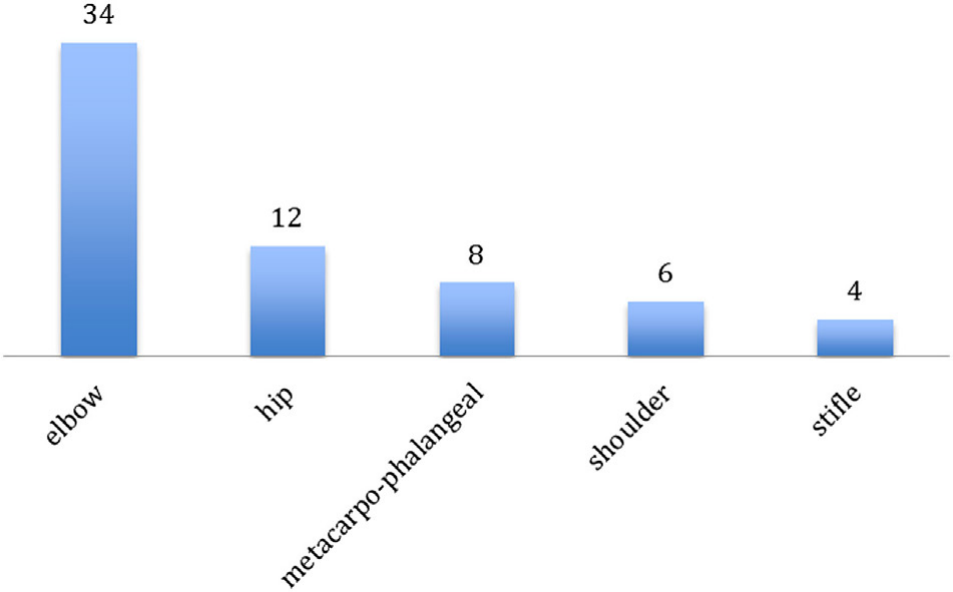
Distribution of 64 joints treated with low-dose radiation therapy in 23 dogs.

64 joints in 25 dogs were treated (Figure 1). The most frequently treated joint was the elbow (*n* = 34; 53%, 14 bilateral, and 6 monolateral), followed by the hip (*n* = 12, 19%, 5 bilateral and 2 monolateral), metacarpophalangeal joints (*n* = 8, 13%, 3 bilateral and 2 monolateral), shoulder (*n* = 6 joints, 9%, all bilateral), and stifle (*n* = 4, 6%, 1 bilateral and 2 monolateral). The number of joints irradiated in the same dog was one (6 dogs), two (8 dogs), three (3 dogs), four (7 dogs), and five (1 dog). Mean anesthesia length was 20 min (range 10–30 min) if 1–3 joints were irradiated and 35 min (range 30–40 min) for the treatment of 4–5 joints.

Because of owner’s logistical reasons, the majority of the dogs (*n* = 21, 84%) were treated over three consecutive days and, only in four cases, the alternate schedule was delivered.

Size of the treatment field was 10 cm × 10 cm (49 joints), 6 × 8 (8 joints), 12 × 12 (6 joints), and 6 × 6 (1 joints). Delivered beam energy was 9 MeV in 3, 12 MeV in 12, and 15 MeV in 49 joints.

### Follow-Up

**Figure 2:**
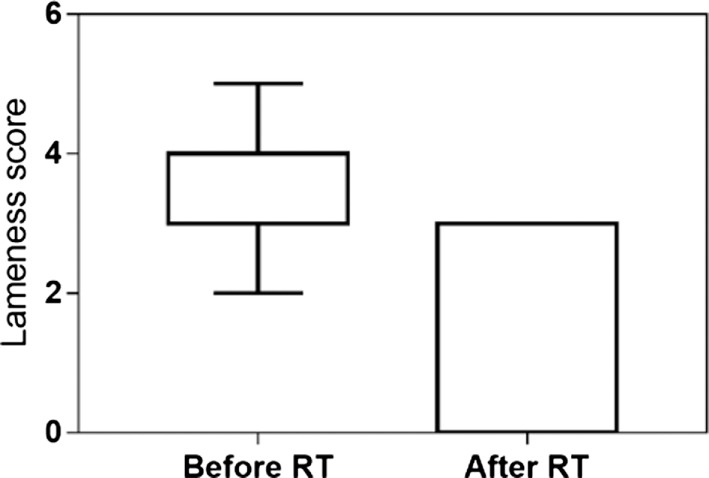
Lameness score evaluated at presentation and 3 weeks after low-dose radiation therapy in 23 dogs. The median lameness score decreased from 4 (range 2–5) to 3 (range 0–3) after RT (*P* < 0.001).

**Figure 3:**
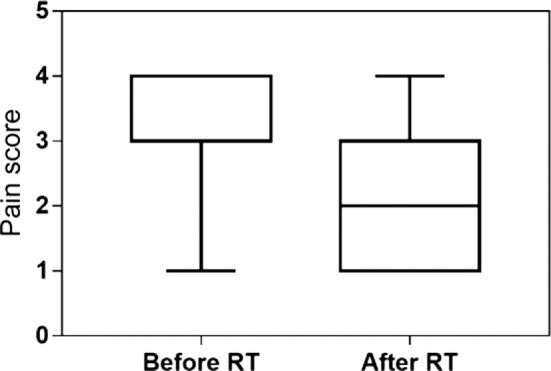
Pain score evaluated at presentation and 3 weeks after low-dose radiation therapy in 23 dogs. The median pain score decreased from 3 (range 1–4) to 2 (range 1–4) after RT (*P* < 0.001).

The clinical recheck 3 weeks after RT was performed in 23 (92%) dogs. At this time, all showed clinical improvement (92%). A partial response was obtained in 16 (64%), and a complete response was documented in 7 (28%) animals. The median lameness and pain scores were, respectively, 4 and 3 before RT and 3 and 2 after RT. Both scores were significantly reduced after the treatment (*P* < 0.001) (Figures 2 and 3). One dog was never rechecked, and the owner reported that there was no improvement after RT. One dog died 2 weeks after the end of RT because of a car accident.

Median follow-up was 437 days (95% CI: 112–762 days). At the end of the study, 21 dogs (84%) had died; in 8 of them, the clinical benefit of RT was still present at the time of death. One dog was euthanized because of OA, whereas 20 dogs died for OA-unrelated causes.

In the 23 dogs experiencing clinical improvement, this lasted for a median of 300 days (95% CI: 104–770). 12 dogs did not receive any medical therapy after RT, whereas medication was administered in 13 dogs after a median of 309 days after RT.

17 dogs (68%) underwent radiographic follow-up of 38 treated joints. The peri-articular soft tissue thickening noted in 33 examined elbows and stifles joint radiographs was subjectively less evident after RT in 22/33 joints (67%), whereas the bony changes were similar.

No acute or late side effects were observed.

**Figure 4:**
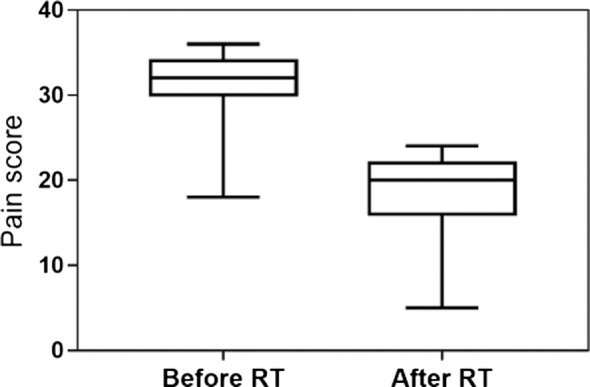
Chronic pain index score obtained from the owner’s questioners before and after RT. The median value changed from 32 (95% CI: 29–33) to 20 (95% CI: 16–20), with a significant decrease after the treatment (*P* < 0.001).

24 owners completed the two questionnaires, 22 of them being satisfied with LDRT because of the improvement of their dogs’ QoL. Before LDRT, the median chronic pain index score was 32 (95% CI: 29–33), whereas it was scored 20 3 weeks after RT (95% CI: 16–20), with a significant decrease after treatment (*P* < 0.001) (Figure 4). Reasons for owners complaint were lack of response (*n* = 1) and short-lasting response (*n* = 1).

### Additional LDRT Treatments

At time of symptoms recurrence, a second course of LDRT was offered to the owners. Four dogs (16%) were re-treated with the same protocol, respectively 218, 266, 39, and 1,384 days after the first treatment. A clinical improvement was observed again and lasted for a median of 418 days (range 60–1,194). One of these dogs, a 7 years old male Labrador Retriever, affected by a severe stifle OA due to cranial cruciate ligament rupture and complications after surgery, received five treatments, with a further clinical benefit of 150, 176, and 173 days. This dog died 48 months after the first LDRT for OA-unrelated causes. At the time of death, the lameness was still under control. Toxicity was not observed in dogs receiving additional LDRT.

Owner consent was obtained for all treated dogs.

## Discussion

Geriatric dogs are often affected by single or multicentric OA, leading to chronic pain and reduced QoL, occasionally leading to the owners’ request for euthanasia. Targeted treatments are often not indicated or possible, and the therapeutic strategies are limited to a combination of drugs and other alternative treatments like nutriceutical and physiotherapy. LDRT is an adjunctive, non-conventional therapy, which can be offered in case of failure of other treatments or if medical therapy cannot be used because of excessive side effects or comorbidities. No data are available in dogs on the use of LDRT delivered to different joints and with a long follow-up.

The present pilot study indicates that LDRT is feasible and useful in dogs with multicentric joint involvement. Indeed, 92% of dogs affected by single or multiple joint OA and irradiated with LDRT showed partial or complete responses after 3 weeks. Notably, the clinical benefit lasted for a median of 300 days, and re-start of medical therapy was considered necessary after a median of 309 days in 13 dogs. All dogs suffered from chronic pain due to OA and previous treatments were insufficient to control the disease, or had to be interrupted because of renal or gastrointestinal side effects.

These results are aligned with previously reported data in human medicine (12–14, 27, 28). Marked and complete pain response has been reported in 58–91% patients irradiated for gonarthrosis, in 24–89% patients with coxarthrosis, and in 63–75% with hand and finger joints OA. Partial and complete response for shoulder and elbow syndrome varied between 58–100 and 63–75%, respectively (13).

In people, the most frequently treated joints are the weight-bearing hip and stifle, suggesting that the involvement of these joints is highly invalidating and requires any effort to be treated (13). Interestingly, 84% of the dogs in the current study were treated for primary forelimb lameness due to shoulder, elbow, and metacarpophalangeal OA or a combination of these conditions, and this may reflect a similar situation, since the paws of the forelimb support the majority of the dog’s weight as demonstrated by temporal–spatial gait analysis (29). This is another element supporting the treatment of multiple joints during the same RT section.

At the time of pain recurrence, LDRT was repeated in four dogs, with a further benefit in chronic pain control for an additional long period (median of 418 days). One dog with severe stifle OA received five LDRT over 48 months, for a total dose of 30 Gy, with a clinical benefit lasting until the dog’s death. In human non-malignant disorders guidelines, a second LDRT is recommended in the case of persisting pain or insufficient pain relief after 6–12 weeks, but treatments beyond a second course are not reported (13, 30). It is important to consider that ionizing radiation has the potential risk of developing secondary neoplasia (31). However, available data in humans indicate that cancer risk after RT for benign diseases of the extremities is very low and comparable to other routine radiologic diagnostic procedures (31). The main factor determining risk is the site of treatment (31). Careful planning is advocated if the treatment field includes a significant amount of red bone marrow (for example, if the shoulder or hips are treated), because of a low (<0.2%) potential risk of leukemia induction (31). Moreover, risk of developing basal cell carcinoma in the irradiated filed has been estimated as 0.006% in people treated with 3 Gy at the extremities (31). Acute or chronic side effects were not observed in this study as well as in dogs receiving multiple LDRT; no dogs developed secondary neoplasia at the treatment site or leukemia.

Recently, the results of a single dose 10 Gy RT treatment in a group of five Labradors affected by elbow OA were reported (21). A short-term benefit was noted: force plate gait analysis before and after RT revealed an improvement in the weight-bearing on the affected limb at weeks 6 and 14. However, the clinical result was less evident in the long period. In the present study, Labrador Retriever and elbow OA were over-represented; 12 elbows in 8 Labrador were treated (4 bilaterally and 4 monolaterally). Lameness grade improved in all but one dog at week 3, and the clinical benefit lasted for a median of 524 days; median pre- and posttreatment chronic pain index was 33 and 18, respectively.

Comparison of the two studies is biased by the different treatment efficacy assessment and different RT protocols. Due to the retrospective nature of the current study, objective force plate analysis was not performed. Efficacy evaluation was based on pre- and posttreatment orthopedic examination (lameness and pain grading) and owner observation through a validated chronic pain questionnaire. Although force plate analysis represents the most objective analysis to document the efficacy of treatment, human guidelines recommend to follow-up patients with physical examinations and subjective pain score assessment during the first year after RT (13, 30). In the current study, dogs were treated with a total dose of 6 Gy delivered in three fractions. There are no established guidelines for RT treatment of small animal OA; however, recommendations for the treatment of painful refractory degenerative joints are available in human medicine, describing a maximum of 6 Gy total dose delivered in small (up to 1 Gy) fractions (27, 30). Based on long clinical benefit documented in the current study, it may be hypothesized that the 3 × 2 Gy protocol is a better choice compared to the single dose 10 Gy treatment. Further prospective studies including a larger group of dogs evaluated with force plate analysis before and after RT are warranted.

In addition to the lack of the force plate analysis, this study has some other limitations, including breed heterogeneity, and inhomogeneous OA causes and severity. Moreover, in 12 dogs, multiple joints were affected and treated. Even if a primarily site of lameness was always identified and objectively rechecked through an orthopedic examination, it is possible that the owner perception of the clinical benefit was influenced by the sum of a multi-centric pain relief. It must be emphasized that the value of the owner’s assessment of chronic pain through a questioners has been shown in veterinary literature (25, 32, 33). On the other hand, some investigations found that owner assessment score improved even in dogs treated with placebo, suggesting a caregiver placebo effect in this outcome measure (34, 35). Therefore, the use of multiple outcome measures combining questioner scores and clinical objective evaluations is recommended in clinical trials studying analgesic treatments for canine OA (36).

Finally, 9–15 MeV electron beam technique could be suboptimal in dose distribution for joints located at depth exceeding 5 cm, especially hips in large dogs and for these cases photon beam RT could provide better dose distribution with a clinical advantage. However, in this study, all dogs with hip OA showed a clinical improvement after RT. Moreover, photon beam RT has the disadvantage that positioning CT and a more time-consuming planning is necessary before RT treatment, with consequent higher costs and longer preparation time.

In conclusion, these preliminary data support the use of LDRT in the management of unresponsive pain due to OA in dogs. Also, LDRT can be repeated without evidence of side effects with prolonged clinical benefit.

## Ethics Statement

This is a retrospective clinical study. Dogs included were treated in accordance with the recommendations of BPV-ANMVI and FVE-UEVP 2015 and approved by written client consent.

## Author Contributions

Study design: PL, SC, and FR; data acquisition: PL, SC, VF, CB, and FR; data analysis and interpretation: PL, SC, VF, CB, and FR; drafting of the manuscript: PL and FR; critical revision and final approval of the manuscript: PL, SC, VF, CB, and FR.

## Conflict of Interest Statement

The authors declare that the research was conducted in the absence of any commercial or financial relationships that could be construed as a potential conflict of interest.
